# C1q as a target molecule to treat human disease: What do mouse studies teach us?

**DOI:** 10.3389/fimmu.2022.958273

**Published:** 2022-08-03

**Authors:** Kristina Schulz, Marten Trendelenburg

**Affiliations:** ^1^Laboratory of Clinical Immunology, Department of Biomedicine, University of Basel, Basel, Switzerland; ^2^Division of Internal Medicine, University Hospital Basel, Basel, Switzerland

**Keywords:** C1q, complement, deficiency, knockout mouse, disease

## Abstract

The complement system is a field of growing interest for pharmacological intervention. Complement protein C1q, the pattern recognition molecule at the start of the classical pathway of the complement cascade, is a versatile molecule with additional non-canonical actions affecting numerous cellular processes. Based on observations made in patients with hereditary C1q deficiency, C1q is protective against systemic autoimmunity and bacterial infections. Accordingly, C1q deficient mice reproduce this phenotype with susceptibility to autoimmunity and infections. At the same time, beneficial effects of C1q deficiency on disease entities such as neurodegenerative diseases have also been described in murine disease models. This systematic review provides an overview of all currently available literature on the C1q knockout mouse in disease models to identify potential target diseases for treatment strategies focusing on C1q, and discusses potential side-effects when depleting and/or inhibiting C1q.

## Introduction

Complement protein C1q was first described in 1961 as the starter molecule of the classical pathway of the complement system. The complement system is an evolutionarily ancient set of about 50 proteins that is critically involved in host defense. An imbalance of complement is known to be related to the development of autoimmunity. Similarly, very rare cases of hereditary C1q deficiency in humans present clinically most notably with the autoimmune disease systemic lupus erythematosus (SLE) or SLE-like symptoms as well as recurrent bacterial infections (1). While this observation supports a protective role of C1q from the development of autoimmunity and infections, there are certain mouse disease models where the absence of C1q has beneficial effects (2–5). In these settings, C1q might function as a target molecule and its manipulation, for example a blockage by small molecules or specific antibodies, could be an effective treatment option. However, C1q is a highly versatile protein. It is well accepted that a number of non-canonical pathways exist: C1q not only triggers the avalanche of protease-activation involved in the classical complement pathway but can also interact directly with receptors thereby affecting numerous cellular processes from proliferation to induction of apoptosis (6, 7). The involvement of C1q is so broad and manifold that it is not straightforward to deduce its role in certain diseases and tissues, let alone the effect of its blockage or absence. Genetically engineered knockout mice provide the opportunity to gain important insights to this question.

C1q is composed of three different chains − A, B and C− which arrange into a hexameric structure resembling a bouquet of tulips (8). In 1998 Botto et al. created a C1qa knockout (C1qKO) mouse with the primary goal to study effects of C1q deficiency on autoimmunity (9). In the C1qKO mouse the expression of C1q is abrogated due to targeted deletion of the *C1qa* gene coding for the A chain that was isolated from the 129/sv genomic library (9). This systematic review provides a comprehensive overview of all studies up to date that employed the C1qKO mouse in the setting of a human disease model. It identifies potential target diseases for therapeutic intervention with C1q as a target molecule, and it illuminates potential side effects, when inhibiting C1q on a systemic level.

## Methods

**Figure 1:**
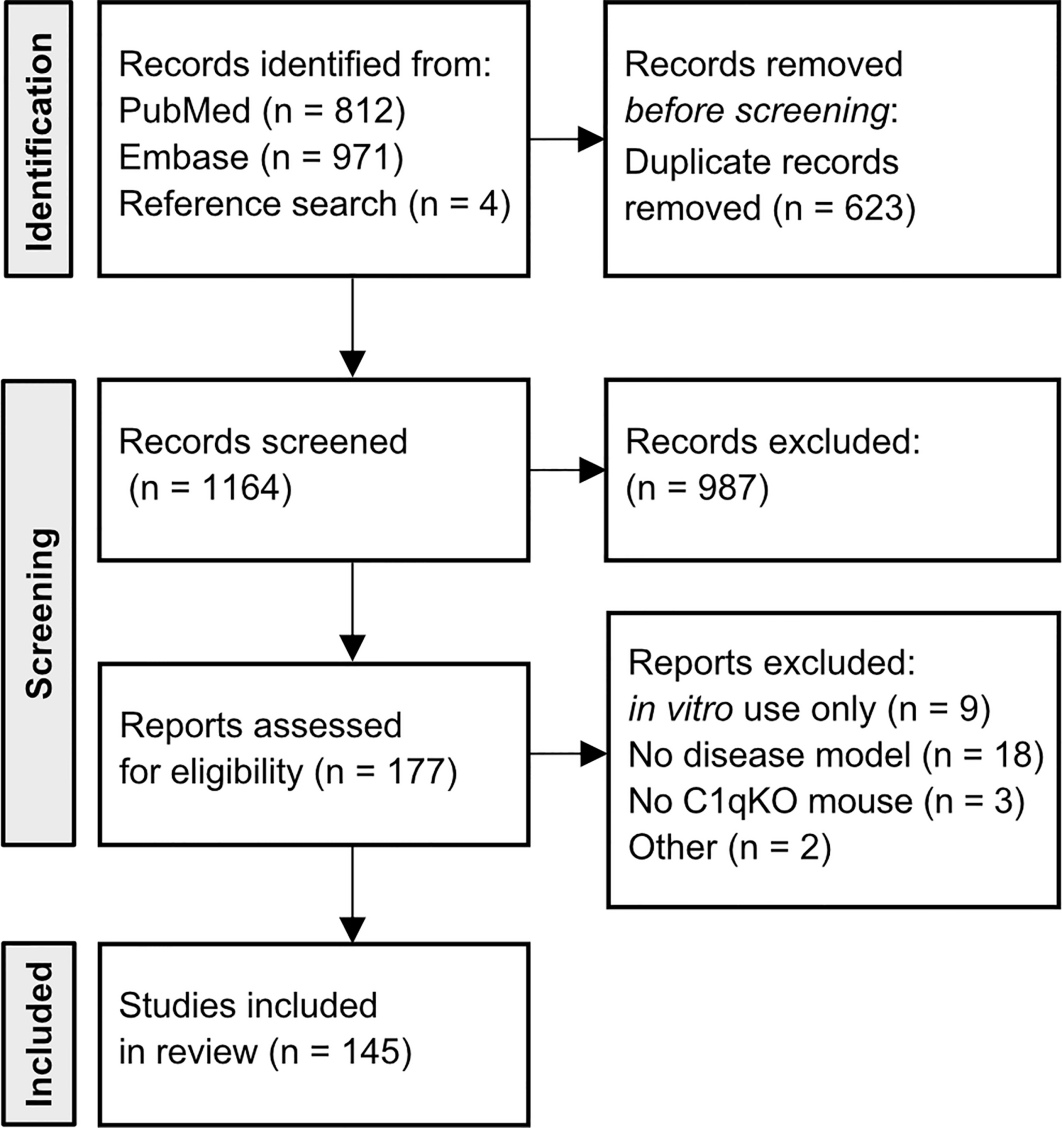
Flow diagram of included articles. Flowchart of number of included articles after database query of PubMed and Embase with search terms “complement C1q”, AND “mouse”. Four articles were included based on reference research of cited articles. Articles were initially screened by title and abstract to meet the inclusion criteria (*in vivo* use of C1qKO mouse, investigated disease model). 177 articles underwent full text review. Thirty-two publications did not fulfill the criteria.

A database query of PubMed and Embase was performed with the search terms “complement C1q” and “mouse”. Broad search terms were chosen to avoid missing relevant publications. Peer-reviewed, PubMed-listed and/or Embase-listed publications written in English and published between 1998 and April 2022 were considered. Embase search was restricted to article type “article”. Removal of duplicates was automated based on DOI number using R software. Titles and abstracts were screened for inclusion criteria, namely usage of the C1qKO mouse in an *in vivo* disease model and/or addressing a human disease in an original research article (**Figure 1**). 177 publications remained for full text review. Criteria for exclusion were *in vitro* use only, no investigation of a human disease model, no use of the C1qKO mouse. One article was excluded due to retraction, another one as it was a case report. In addition, references of included studies were screened for relevant publications yielding four additional studies. In total, the databank search resulted in 145 publications. Publications were grouped according to disease mechanism and organ manifestation, respectively, yielding nine clusters. Each publication was evaluated with respect to disease outcome of the C1qKO mouse as compared to wild type (wt) control (**Tables 1**–**3**).

**Table 1:** Overview of publications in the disease clusters CNS/PNS and retina, Ischemia and reperfusion and Liver diseases.

Disease entity	oc	Disease model	Gene manipulation and genetic background	Sex	Ref
**CNS/PNS and retina**					
**Retina**	-	pruning/glaucoma*	DBA/2J	f/m	(10)
	+	glaucoma	DBA/2J	f	(11)
	+	glaucoma	DBA/2NNia	f/m	(12)
	+	glaucoma	DBA/2J	f/m	(13)
	=	AMD	rd1	f/m	(14)
	=	AMD	C1q^-/-^, C1q^-/-^*MBL^-/-^*	f/m	(15)
	=	AMD		f/m	(16)
	+	AMD		f/m	(17)
	-	retinal aging		f/m	(18)
**Neuro-degeneration and aging**	+	AD	Q^-/-^, APPQ ^-/-^, APPPS1Q^-/-^, C57BL/6, B6/SJL	f/m	(2)
	+	AD	APPQ ^-^/^-^, APPPS1Q^-^/^-^, B6/SJL	f/m	(19)
	=	AD	3xTgBUBC1q^-^/^-^, BUB/BnJ	f/m	(20)
	+	AD		f/m	(21)
	+	FTLD	*Grn^-^/^-^;C1q^-^/^-^*	f/m	(22)
	+	FTLD	*Grn^-^/^-^;C1q^-^/^-^, Grn^-^/^-^;C1q^-^/^-^;C3^-^/^-^*	f/m	(23)
	=	ALS	SOD1^G37R^/C1q^-^/^-^	f/m	(24)
	+	ALS	*IL-1α^-/-^ TNFα^-/-^ C1q^-/-^*, *IL-1α^-/-^ TNFα^-/-^ C1q^-/-^ SOD1^G93A^*	f/m	(25)
	=	M. Parkinson		m	(26)
	-	amyloid neuropathy	mTTR^-/-^hTTR^Met30+/+^mC1q^-/-^, 129X1/SvJ/C57BL/6	f/m	(27)
	+	OIBP		f/m	(28)
	+	brain aging		f/m	(29)
**CNS injury**	=	TBI		f/m	(30)
	+	TBI	*C1q^-/-^, IL-1α^-/-^ Tnf^-/-^ C1q^-/-^*	f/m	(31)
	-	TBI		f/m	(32)
	-	TBI		f/m	(33)
	+	TBI		f/m	(34)
	+	injury by radiation	*C1qa^FL/FL :^ Cx3cr1^CreERT2/WganJ^*	m	(35)
	+	spinal cord injury	BUB/BnJ	m	(36)
	=	spinal cord injury	BUB/BnJ	m	(37)
**PNS injury**	=	peripheral nerve lesion		f	(38)
	=	peripheral nerve lesion		f/m	(39)
**Infection**	+	prion disease	*C1qa^-/-^*, *C1qa/H2-Bf/C2^-/-^*	f/m	(3)
	+	prion disease		f	(4)
	=	prion disease		f/m	(40)
	=	HIV-and HAND		f/m	(41)
**MS**	+	MS		f/m	(42)
	=	MS		f/m	(43)
	+	MS	*C1q^fl/fl^;TMEM-Cre^ERT/+^*	f/m	(44)
**Depression**	-	depression		m	(45)
**Epilepsy**	-	epilepsy		f/m	(46)
	-	epilepsy	background n.s.	m	(47)
**Ischemia and reperfusion**					
**H/I**	+	H/I stroke		f/m	(5)
	+	H/I stroke		f/m	(48)
**Stroke**	=	ischemic stroke		f/m	(49)
	=	ischemic stroke		m	(50)
	+	ischemic stroke	C1q/MBL^-/-^	m	(51)
**I/R**	+	retinal I/R		f/m	(52)
	=	GI I/R		m	(53)
	=	GI I/R		f/m	(54)
	+	GI I/R		f/m	(55)
	+	myocardial I/R	C1q^-/-^, C1q/fD^-/-^	m	(56)
	+	skeletal muscle I/R		f/m	(57)
	=	cutaneous I/R		m	(58)
**Liver diseases**					
**Liver toxicity**	+	ALD		f	(59)
	+	ALD	*C1qa^-/-^*, *C1qa/FD^-/-^*	f	(60)
	+	hepatotoxicity		m	(61)
	=	hepatotoxicity		f	(62)
	+	NASH		m	(63)

Each cluster is subdivided according to disease and organ manifestation, respectively. Disease outcome (oc) of C1qKO mice compared to wt and/or C1q sufficient mice in the investigated disease model is given as “+” respectively turquoise =beneficial, “-” respectively ocher =detrimental, “=“ respectively grey=no effect. The overall outcome on the disease entity is similarly color coded using lighter shades for ambiguous group results. Genetic modifications other than C1qKO and genetic background other than C57BL/6 are listed explicitly. In studies with several C1q deficient mice, all C1q deficient mice are listed. Sex as indicated in the study (f=female only, m=male only, f/m=mixed gender); if not mentioned explicitly by the study, mixed gender was assumed. AD, Alzheimer’s disease; ALD, alcoholic liver disease; ALS, amyotrophic lateral sclerosis; AMD, age-related macular degeneration; APP, amyloid precursor protein; FLTD, frontotemporal lobar degeneration; GI I/R, gastrointestinal ischemia/reperfusion; H/I, hypoxia/ischemia; HAND HIV, associated neurocognitive disorder; I/R, Ischemia/reperfusion; MS, multiple sclerosis; NASH, non-alcoholic steatohepatitis; n.s., not specified; OIBP, obesity induced brain pathology; PNS, peripheral nervous system; TBI, traumatic brain injury. *in the Glaucoma part of the study no C1qKO mouse model was used; the outcome classification relates to synapse elimination.

**Table 2:** Overview of publications in the disease clusters autoimmunity and infectiology.

Disease entity	oc	Disease model	Gene manipulation and genetic background		Sex	Ref
**Immunology**						
**Autoimmunity**	-	C1qKO induced SLE	129/Ola, 129/Ola×C57BL/6 F2		f/m	(9)
	-	C1qKO induced SLE	129/Sv, 129/Sv×C57BL/6 F2		f/m	(64)
	-	C1qKO induced SLE			f/m	(65)
	-	C1qKO induced SLE	129/Sv, 129/Sv×C57BL/6, C57BL/6		f/m	(66)
	=	C1qKO induced SLE	*C1qa-/-* Ig^HEL^, *C1qa-/-* Ig^HEL^/sHEL		f/m	(67)
	-	C1qKO induced SLE	129/Ola×C57BL/6		f/m	(68)
	-	C1qKO induced SLE	129/Sv×C57BL/6, C57BL/6		f/m	(69)
	=	C1qKO induced SLE			f/m	(70)
	-	C1qKO induced SLE	C57BL/6.*C1q^-/-^*, C57BL/6.*lpr/lpr*.*C1q^-/^*^-^, MRL/Mp-*lpr/lpr*.*C1q^-/-^*		f/m	(71)
	-	C1qKO induced SLE	MRL/Mp.*C1q^-/-^*,		f/m	(72)
	-	Cq1KO induced SLE	129×B6 F2		f	(73)
	-	Cq1KO induced SLE	C1q^-/-^Ig^HEL^, C1q^-/-^Ig^HEL^/mHEL-KK		f/m	(74)
	=	Cq1KO induced SLE	V_H_3H9R/V_L_κ8R.MRL/Mp.*C1qa^-/-^*, V_H_3H9R.MRL/Mp.*C1qa^-/-^*		f/m	(75)
	+	pristane induced SLE	BALB/c		f	(76)
	-	Cq1KO induced SLE			f	(77)
	-	Cq1KO induced SLE			f	(78)
	-	autoimmunity	MRL/Mp.*C1q^-/-^*, C57BL/6.*C1q^-/-^*		f	(79)
**Autoimmune nephropathy**	-	LN	*C1qa^-/-^*, *C1qa/H2-Bf/C2^-/-^*, 129/Sv×C57BL/6		f/m	(80)
	-	LN	*Sle1.C1q^-/-^, Sle1.Mfge8^-/-^C1q^-/-^*		f/m	(81)
	=	LN			m	(82)
	-	Anti-GBM GN	C1qa^-/-^, C1qa/H2-Bf/C2^-/-^, 129/Sv×C57BL/6		f/m	(83)
	-	Anti-GBM GN	129/Sv×C57BL/6, C57BL/6		f/m	(84)
	=	Anti-GBM GN			f/m	(85)
	=	Cryoglobulinemic GN	BALB/c		f/m	(86)
	=	FSG sclerosis	BALB/c		f	(87)
	=	tubulointestinal fibrosis			m	(88)
**Transplant rejection**	-	transplant at rejection			f	(89)
	-	transplant at rejection	C57BL/6, BALBc		f	(90)
	-	transplant at rejection			f	(91)
**Arthritis**	=	arthritis	*C1q^-/-^*, *C1q^-/-^/MBL^-/-^*		f/m	(92)
	+	arthritis	*C1q^-/-^/Df^-/-^*		f/m	(93)
	=	anaphylaxis	129/SV		f/m	(94)
**Vaccination**	=	rhesus prophylaxe			f/m	(95)
	+	immunoprophylaxis				(96)
	=	HSV- Impfung			f	(97)
	=	adenoviral vectors			f/m	(98)
	=	adenoviral vectors			m	(99)
	-	IBD	C1q/MBL^-/-^		f/m	(100)
	+	sterile inflammation	C1q^-/-^, C1q/fD^-/-^		f/m	(101)
**Infectiology**						
**Bacterial infections**	-	S. Pneumoniae			f/m	(102)
	-	S. Pneumoniae			f/m	(103)
	-	S. Pneumoniae septicaemie			f/m	(104)
	-	S. Pneumoniae meningitis			f/m	(105)
	-	S. Pneumoniae acute otitis			f/m	(106)
	-	S. Pneumoniae acute otitis			f/m	(107)
	-	S. Pneumoniae acute otitis			f/m	(108)
	-	S. Pyogenes septicaemie			f/m	(109)
	-	polymicrobial peritonitis	129/SV		m	(110)
	-	polymicrobial peritonitis	129/SV		f/m	(111)
	-	polymicrobial peritonitis			f/m	(112)
	-	Salmonella enterica	129/SV		m	(113)
	-	Borrelia burgdorferi			f/m	(114)
	-	Rickettsia australis			f/m	(115)
**New therapeutica**	-	N. gonorrhoeae			f	(116)
	-	N. gonorrhoeae			f	(117)
	=	N. gonorrhoeae			f	(118)
	=	P. aeruginosa			m	(119)
**Other pathogens**	-	West Nile virus	C1q^-/-^, C1q×fD^-/-^		f/m	(120)
	-	Malaria	129/Sv		f	(121)
	=	Nematode	–		f/m	(122)
	=	Cryptosporidium	–		f/m	(123)
	=	Candida albicans	–		f/m	(124)

Each cluster is subdivided according to disease and organ manifestation, respectively. Disease outcome (oc) of C1qKO mice compared to wt and/or C1q sufficient mice in the investigated disease model is given as “+” respectively turquoise =beneficial, “-” respectively ocher =detrimental, “=“ respectively grey=no effect. The overall outcome on the disease entity is similarly color coded using lighter shades for ambiguous group results. Genetic modifications other than C1qKO and genetic background other than C57BL/6 are listed explicitly. In studies with several C1q deficient mice, all C1q deficient mice are listed. Sex as indicated in the study (f=female only, m=male only, f/m=mixed gender); if not mentioned explicitly by the study, mixed gender was assumed. FSG, focal segmental glomerulosclerosis; GBM, glomerular basement membrane; GN, Glomerulonephritis; HSV, herpes simplex virus; IBD, inflammatory bowel disease; LN, lupus nephritis; N., Neisseria; P., Pseudomonas aeruginosa; S., Streptococcus; SLE, systemic lupus erythematosus.

**Table 3:** Overview of publications in the disease clusters Vascular diseases, Pregnancy, Cancer and Various.

Disease entity	oc	Disease model	Gene manipulation and genetic background	Sex	Ref
**Vascular diseases**					
**Atherosclerosis**	-	atherosclerosis	*C1q^-/-^/Ldlr^-/-^*	f	(125)
	-	atherosclerosis	*C1q.Ldlr^-^/^-^*, *C1q.sIgM.Ldlr^-^/^-^*	f	(126)
	-	woundhealing	–	f/m	(127)
	-	ALI	–	f	(128)
	-	primary hemostasis		f/m	(129)
**Pregnancy**					
**Fetal loss**	-	fetal loss		f	(130)
	+	fetal loss	C1q/fD ^-/-^	f	(131)
	=	fetal loss		f	(132)
**PE**	-	PE		f	(133)
	-	PE		f	(134)
	-	PE		f	(135)
**Cancer**					
**Solid tumor**	+	melanoma		f/m	(136)
	=	breast cancer	neuT^+^-C1q^-^/^-^, BALB/c	f	(137)
	+	ccRCC		f/m	(138)
**Tumor therapy**	-	immunotherapy		f/m	(139)
	=	immunotherapy		f/m	(140)
	=	immunotherapy		m	(141)
	-	immunotherapy	SCID/C1q^-/-^	f	(142)
**Various**					
**Skin**	=	burn injury		f/m	(143)
	+	epidermolysis bullosa	BALB/c	f/m	(144)
	+	muscle regeneration		f/m	(145)
**Pulmo**	-	COPD		f	(146)
	+	pulmonary fibrosis		f/m	(147)
	=	AA amyloidosis	C57B L 6×129/SV	f/m	(148)
	=	adipose inflammation		f	(149)

Each cluster is subdivided according to disease and organ manifestation, respectively. Disease outcome (oc) of C1qKO mice compared to wt and/or C1q sufficient mice in the investigated disease model is given as “+” respectively turquoise =beneficial, “-” respectively ocher =detrimental, “=“ respectively grey=no effect. The overall outcome on the disease entity is similarly color coded using lighter shades for ambiguous group results. Genetic modifications other than C1qKO and genetic background other than C57BL/6 are listed explicitly. In studies with several C1q deficient mice, all C1q deficient mice are listed. Sex as indicated in the study (f=female only, m=male only, f/m=mixed gender); if not mentioned explicitly by the study, mixed gender was assumed. ALI, acute lung injury; AA, amyloid A protein; COPD, chronic obstructive pulmonary disease; PE, preeclampsia.

## Results

### 1. C1q deficiency and diseases of the brain and retina

Most studies showing beneficial effects of C1q deficiency were performed in the immune-privileged brain and retina (**Table 1**). This is the disease cluster with the largest number of studies using the C1qKO mouse and a remarkable increase in publications in recent years.

#### 1.1 C1q deficiency and neurodegenerative diseases of the retina

In 2007, Stevens et al. showed for the first time that C1q is involved in synaptic pruning in the developing brain as well as in neurodegeneration in the visual system, a prime system for studies on synaptic refinement (10). In the postnatal brain, retinal ganglion cells (RGC), which form the optic nerve with their axons, expressed C1q in the presence of immature astrocytes. C1q mainly localized to immature synapses in the downstream thalamic dorsolateral geniculate nucleus (dLGN). The physiological relevance of C1q for retinogeniculate refinement became apparent in C1qKO mice, that presented with reduced eye-specific segregation of RGC input onto dLGN. Knockout of the downstream complement component C3 in C3 knockout (C3KO) mice presented with a similar phenotype supporting complement activation as the underlying mechanism involved in this setting of synaptic pruning (10). Phosphatidylserine, which might act as a synaptic tag and serve as an “eat-me” signal, was elevated in the dLGN of C1qKO mice (150).

In glaucoma, neurons of the retina undergo neurodegeneration induced by elevated intra-ocular pressure. The DBA/2J mouse is a reproducible murine glaucoma model. Though not employing the C1qKO mouse in this section of the study, Stevens et al. provided evidence for C1q mediated synapse loss in the retina as an early crucial event in glaucoma, preceding axonal and thus optic nerve damage (10). In summary, this study demonstrated a prominent role of C1q in synapse elimination in the visual system during development as well as in the diseased state of neurodegeneration (10)

A number of studies of C1qKO mice confirmed detrimental effects of C1q in early stages of glaucoma (11–13) (**Table 1**). C1qKO mice showed markedly reduced optic nerve damage, an effect that faded with age (11). Early synaptic and dendritic atrophy of RGC was abolished in C1qKO mice and similarly could be preserved using pharmacological inhibition (13). A detailed study by Kumari et al. confirmed these results and identified a sex-dependency: only female C1qKO mice on a DBA/2NNia background had elevated intra-ocular pressure (12). The detailed time analysis supported the idea that the protective effect of C1q deficiency was restricted to early glaucoma and lost as the disease progressed (12). Taken together, these studies suggest C1q as a potential target only in very early stages of glaucoma before apparent axonal loss and thus clinical signs.

The effects of C1q deficiency on age-related macular degeneration (AMD) are less uniform (**Table 1**). AMD has a multifactorial genesis with polymorphisms in the complement system being one of the risk factors (151). The dry form is dominated by drusen and may transition to the wet form, characterized by an unfavorable increase in neovascularization. Photo-oxidative damage as a model for AMD showed a better long-term outcome in C1qKO mice (17). In particular microglia and macrophages had reduced levels of inflammasome activation (17). In contrast, several other models of AMD showed no influence by C1q deficiency. The rd1 mouse with a recessive mutation in the phosphodiesterase gene, is a model system to study photoreceptor degeneration and was employed to mimic early changes in AMD marked by rod cell death (14). Although C1q was highly expressed in retinas undergoing degeneration in the rd1 mouse, C1q deficiency affected neither the progression of the disease nor the clearance of photoreceptor-debris (14). Similarly, there was no clear effect of sole C1q deficiency in a model for wet AMD by laser-induced choroidal neovascularization (15). Unwanted neovascularization appeared to require complement amplification by the alternative pathway, as double knockout of the classical as well as the lectin pathway by C1qKO and mannose-binding lectin (MBL)KO caused equal protection as a factor B knockout (15). Also inflammation and microglial activation induced by immune complex-formation in the retina, a phenomenon associated with early AMD, remained unchanged in the C1qKO mouse (16).

Lastly, while C1q deficiency seems to be protective for neurodegenerative diseases of the retina, it aggravates retinal thinning during normal aging. This indicates that C1q and other complement components are important for retinal homeostasis during aging (18).

#### 1.2 C1q deficiency and neurodegeneration

Brain C1q protein levels increase with age: while Stephan et al. reported a dramatic 300-fold increase in C1q protein levels detected by immunofluorescent signal in tissue slides of the aging mouse brain (29) other studies using novel monoclonal antibodies reported a moderate, less than two fold rise in brain homogenate (152). Normal aging brain was beneficially influenced by C1q deficiency, though effects on synaptic plasticity and memory were small and inconsistent across age-groups (29).

While the physiologically aging brain is known to show some functional constraints, one needs to distinguish a pathological loss of neurons occurring during neurodegeneration. The complement system is by now well accepted to have an influential role in this disease entity (153). Neurodegeneration is associated with neuronal cell death, synaptic loss, and neuroinflammation. Microglia and reactive A1 astrocytes are important cellular actors for all of these processes and both are intimately linked to C1q (31). Microglia, the local tissue macrophages, are a major source of C1q in the brain (154). Astrocytes, star-shaped glial cells, traditionally have mainly supportive functions for neuronal metabolism and homeostasis, but can lose these features in the reactive A1 state. C1q is one of the factors which turn homeostatic functioning astrocytes into the reactive A1 state (31).

*Alzheimer’s disease* (AD) is a prime example of a neurodegenerative disease histologically characterized by extracellular ß-amyloid plaques and intracellular tau tangles. Common murine models use genetic modifications leading to altered expression of the relevant proteins amyloid precursor protein (APP), presenilin 1 or 2 and/or tau. C1q has been shown to co-localize with ß-amyloid plaques (155). C1q deficiency did not alter plaque formation, but drastically reduced astrocytic re-activity in the vicinity of plaques causing reduced neuronal injury and thus improved neuronal integrity (2). Furthermore, ß-amyloid induced synapse loss was dependent upon C1q: intracerebral injection of oligomeric ß-amyloid did not induce synapse loss in C1qKO mice and, similarly, synapse loss could be prevented by concomitant application of an anti-C1q antibody (21). As expected, C1q levels in C1qKO APP mouse brains were undetectable. However, C3 levels were high both intracellularly, due to their expression by astrocytes, and extracellularly as the cleavage products C3b/iC3b on fibrillary amyloid plaques (19). It was reasoned that C3– probably activated *via* the alternative pathway– acted neuroprotectively (19). In contrast, another study showed neuroprotective effects of C1q *via* CREB induced low density lipoprotein receptor-related protein 1B (LRP1B) and G protein-coupled receptor 6 (GPR6) expression *in vitro* (20). LRP1B and GPR6 levels in young animals were significantly lower in C1q deficient 3xTgBUB Alzheimer mice. It was inferred that C1q acts neuroprotectively during the early phases (pre-plaque formation) of the disease, while detrimental effects of C1q occur *via* co-expression of C1r and C1s in late stages (20). However, the expression pattern of C1q only paralleled LRP1B and GPR6 levels in a very limited degree with levels most prominently elevated at >13 months of age (20). Thus, effects of C1q on AD may be complex and age dependent.

*Frontotemporal lobar degeneration* (FLTD) is a neurodegenerative disorder with symptoms most strikingly encompassing personality changes with blunting of emotions as well as the development of aphasia. The familiar form is known to be related to mutations in the progranulin gene (GRN), which causes protein aggregation. In the murine genetic model for FLTD using progranulin deficiency, two considerable studies showed C1q deficiency induced reduced neurotoxicity and synaptic pruning by microglia (22, 23). Importantly, C1q deficiency also mitigated phenotypical obsessive compulsive behaviors and premature mortality (22). Microglia, expressing high levels of the causal protein progranulin as well as lysosomal and complement genes, were the key players to induced synaptic pruning of preferentially inhibitory synapses in the ventral thalamus (22) and transitioned to an activated state in the GRN-knockout mouse model (23). Microgliosis was mitigated in double GRN- and C1qKO mice and near-completely rescued in the triple knockout for GRN, C1q, and C3. Similarly, pharmacological blocking of the complement membrane attack complex reduced neuronal cell death (23). Thus, C1q-mediated complement activation may underlie microglial transmitted neuronal cell death in this disease model.

*Morbus Parkinson* is characterized by a loss of dopaminergic neurons of the substantia nigra, which causes typical movement related symptoms like tremor, rigidity, and slowed gait but also cognitive deficits. There was no evidence for an effect of C1q deficiency on loss of nigral dopaminergic neurons, striatal dopaminergic fibers or dopamine levels in the murine Parkinson’s disease model by 1-methyl-4-phenyl-1,2,3,6-tetrahydropyridine (MPTP)-induced loss of nigral dopaminergic neurons (26). There is currently no study investigating potential behavioral differences also over a prolonged disease course.

*Amyotrophic lateral sclerosis* (ALS) is a severe neurodegenerative disorder characterized by a rapidly progressing loss of primary and secondary motor neurons leading eventually to death due to respiratory motor failure. In a murine disease model expressing mutated superoxide dismutase (SOD1), C1q deficiency alone only caused histological improvement such as decreased synaptic loss of cholinergic nerve terminals onto motor neurons, while leaving the clinical outcome and progression of the disease unaffected (24). However, the triple knockout of reactivating astrocytes A1 inducing factors Il-1α,TNFα and C1q affected gliosis positively leading to a marked extension of mouse survival (25).

Obesity-induced brain pathology is characterized by typical cerebrovascular and white matter signs, which are related to microglia phagocytosis. These are not present in obese C1qKO mice under a western diet (28).

In a model for amyloid neuropathy caused by aberrant transthyretin (TTR), amyloid depositions were exacerbated in C1qKO mice most likely due to decreased phagocytosis (27). Thus, in the field of neurodegenerative studies, this is the only clear exception to else overall beneficial effects of C1q deficiency (**Table 1**).

#### 1.3 C1q deficiency and brain injury

Upon brain tissue damage, astrocytes are known to be reactivated and can form glial scar tissue. Liddelow et al. explored C1q involvement in the formation of reactive astrocytes by microglia (31). The release of C1q, Il-1α and TNFα from activated microglia triggered the formation of reactive astrocytes. A1 astroctyes lost their ability to support neurons and induced death of axotomized neurons in a model of optic nerve crush (31). A1 astrocytes are also of interest with respect to the above explored neurodegenerative diseases as A1 astrocytes – positive for C3 – have been shown to be present in human post-mortem tissue from a large number of neurodegenerative and neuroinflammatory diseases (31). While C1qKO mice still had the ability to form A1 astrocytes, this was strongly reduced in Il-1α and TNFα double knockouts and completely blocked in triple knockouts. With respect to optic nerve crush, RGC remained intact seven days after intervention in double and triple knockout mice as opposed to wt mice (31). As the effect was already present in the double knockout, the role of C1q for this outcome might be weak. Intravitreous treatment of wt mice with anti-C1q antibodies altered astroglia expression pattern only slightly, while the combination of anti-C1q, anti-TNFα and anti-Il-1α hindered A1 formation (31). In contrast, a different study showed that the treatment success of RGC axon regeneration after optic nerve injury critically depended upon the presence of C1q: While C1q deficiency alone did not change axon regeneration, it significantly reduced the regeneration in the presence of various pro-regenerative treatments (33). The route of delivery was critical for effects on regeneration: Anti-C1q antibody treatment was only hindering regeneration when delivered intranervally or systemically but not intravitreously. The authors inferred that blocking C1q and/or the absence of C1q caused reduced removal of myelin, with myelin being a growth-inhibitor for axon regeneration (33). Similarly, postinjury debris clearance was reduced in C1qKO as well as microglia-deficient Itgam KO mice when examining the dLGN after optic nerve crush, a setting of Wallerian degeneration (32). In contrast to Liddelow et al., C1q deficiency had no protective effect on RCG cell survival in this study (32). Thus, while data from the Liddelow et al. study point to a protective effect of C1q deficiency on RCG cell survival (31), Peterson et al. looked beyond effects on the retina itself and observed hampered axon regeneration in the absence of C1q (33)

In a recent study, a clear effect of C1q on secondary long term injury after traumatic brain injury (TBI) by cortical impact could be demonstrated: neuroinflammation and cell loss in the thalamus three weeks following injury in sensoricortex was markedly reduced in C1qKO mice and was similarly demonstrated by repetitive intraperitoneal (i.p.) application of anti-C1q antibodies (34). However, functional effects as measured on a shorter timescale (1-7 days post injury) by memory tests and motor performance were not present in C1qKO as well as C3KO mice undergoing a similar brain injury protocol; only C4 knockout (C4KO) mice presented with reduced motor deficiency (30).

Mice with flox-targeted absence of C1q in microglia undergoing cranial radiation induced brain injury showed reduced neuroinflammation and reduced synaptic loss when compared to wt mice (35). They presented clinically without cognitive deficit (35).

Outcomes in spinal cord injury were not uniform: while lesion volume was reduced in the C1qKO mice after spinal cord contusion (36) it was unchanged to wt after transection (37). Functionally there was a small but significant increase in fine locomotor recovery in C1qKO mice (36). In cell culture, C1q increased neurite growth by inhibiting repulsive signaling of myelin associated glycoprotein (MAG). Axons of C1qKO mice expressed increased turning after dorsal column transection but no gross effects on total sprouting or midline crossing were noted (37). The consequence of turning with respect to sensorimotor function remained unexplored (37).

In contrast to the central nervous system (CNS), there is no evidence so far for a crucial role of C1q in the peripheral nervous system (PNS): there was neither a difference in motor recovery or cell survival in unilateral facial nerve crush (39) nor in behavioral motor outcome and synaptic terminals of affected motoneurons by sciatic nerve crush (38). Synapse elimination in the PNS may instead involve Schwann cells and complement independent mechanisms (156).

#### 1.4 C1q deficiency and epilepsy

Synaptic pruning, the process of synapse elimination of excessive synapses in the neonatal brain, occurs throughout the mammalian brain and is known to continue until early adulthood in humans. As mentioned above, in C1q deficient mice, synaptic pruning was disturbed and associated with dysfunctional synaptic refinement in the dLGN (10). Excessive glutamatergic inputs and epileptogenic activity in C1qKO mice with a behavioral phenotype of atypical absence seizure seen as “freezing” behavior was reported and would be in line with failed synaptic pruning on brain wide neuronal circuitry (46). Microscopic analysis confirmed anatomical changes, such as higher spine density on basal dendrites of layer Vb neurons in the sensorimotor cortex, which is most likely due to inadequate synaptic pruning (47). However, there is also evidence that the role of C1q in synaptic pruning cannot be generalized to all parts of the brain: C1q deficiency had neither an influence on synaptic pruning and development of normal hearing in the auditory system (157) nor on the primary visual cortex (158). With respect to epilepsy, electroencephalography (EEG) recordings were performed postnatally with the oldest animal being 60 days of age (46), and it remains unclear, whether epileptic EEG patterns and behavioral abnormalities persist in later adulthood. Similarly, while C1q is needed for synaptic refinement in dLGN during development (10), it is not required for later plasticity in the ocular dominance sensitive phase in primary visual cortex (159). Thus, it is possible that the effects of C1q deficiency are dependent on the developmental stage and the specific brain regions involved.

Although, a predisposition to epileptic seizures is not uncommon in inbred mouse strains (160), the occurrence of epilepsy in C1qKO mice has to be taken into account when interpreting results in other areas, in particular brain-connectivity related diseases like neurodegeneration, which might potentially be influenced by ongoing epilepsy.

From a translational point of view it is worth mentioning that, in contrast to the mouse model, neuropsychiatric lupus and epileptic seizures are a rather uncommon finding in C1q deficient humans with SLE (1, 161).

#### 1.5 C1q deficiency and infectious diseases of the brain

The CNS is an immune privileged system: The blood brain barrier forms a border to the systemic circulation allowing only certain molecules and cells to enter the brain. Tissue grafts show prolonged survival, foreign antigens do not readily elicit an immune response.

In pneumococcal meningitis in complement deficient mice (C3 and C1qKO), a remarkable differential regulation of cytokines with systemic up-regulation and concomitant down-regulation in the CNS was observed (105). The down-regulation was associated with fewer intracranial complications such as intracranial pressure elevation and blood brain barrier disruption. However, overall there was a pronounced lethal outcome in the C1qKO mouse that was related to secondary blood spread and systemic actions (105).

In human immunodeficiency virus associated neurocognitive disorder synapse loss was accompanied by C1q depositions (41). However, C1q was not causally related, as knockout of C1q did not rescue synapse loss (41).

Prion diseases are a group of rare transmissible brain diseases with the infectious agent being an abnormally folded prion protein, which induces misfolding of their naturally occurring correctly folded counterpart cellular prion protein (PrP^C^). Amyloid aggregates of misfolded proteins and vacuoles are micro- and macroscopic hallmarks of lethal prion diseases such as scrapie in sheep, bovine spongiform encephalopathy in cattle or Creutzfeldt-Jakob disease in human. The process of neuroinvasion, i.e. entering of the pathogen into the brain, was massively hampered in C1qKO mice (3, 4). While prion inoculation into brain tissue led to development of scrapie in all tested mice, peripheral i.p. inoculation resulted in markedly delayed disease expression (4) and did not cause pathological changes in C1qKO mice when only limited prion titres were inoculated (3). Mechanistically, antigen uptake by dendritic cells was affected. Thus, neuroinvasion of peripherally applied prion requires C1q, a transporter function that can be overcome by a high load of prion inocula.

The role of naturally abundant PrP^C^ is still less clear. Splenic PrP^C^ is upregulated in response to an immunological challenge by immune complexes or stomatitis virus, a C1q dependent effect which is absent in C1qKO mice (40).

#### 1.6 C1q deficiency and inflammatory autoimmune diseases of the brain

Multiple sclerosis (MS) is an inflammatory autoimmune disease of the CNS with destruction of myelin sheaths. A widely used model to induce MS like symptoms in mice is the myelin oligodendrocyte glycoprotein (MOG) -experimental autoimmune encephalomyelitis (EAE) model, whereby MOG peptide is administered subcutaneously. C3 deficiency attenuated disease severity measured in a number of behavioral read outs, while lack of complement C1q *per se* did not change the disease course (43). However, flares, commonly seen after anti-MOG antibody application, were abolished in this model of MS in C1qKO mice (42). Microglia-targeted knockdown of C1q in an EAE model reduced microgliosis (44). Again, the clinical progression of the disease and clinical scoring remained unchanged. Interestingly, the application of a specific C1q blocking antibody (ANX-M1.21) reduced IBA1-microglia/macrophages in the tested hippocampal region similarly, arguing for anti-C1q antibodies to potentially inhibit chronic inflammation in white matter (44).

#### 1.7 C1q deficiency and depression

C1qKO mice show a pronounced learned helplessness behavior in response to an inescapable foot-shock paradigm, a well-established model to induce depressive-like behavior (45). Wt mice showed reduced C1q mRNA levels in the prefrontal cortex (PFC) in response to foot-shocks as compared to unshocked mice. Interestingly, levels of pro-inflammatory cytokines in the PFC were significantly increased in C1qKO mice independent of the shock paradigm (45). It remains uncertain, whether these increased cytokines relate to increased levels as seen in post-mortem brain samples of patients or merely reflect an altered balance in the C1qKO brain.

### 2. C1q deficiency and ischemia and reperfusion (I/R) injury

In diseases like stroke or myocardial infarction a period of hypoxia caused by e.g. a thrombus obviating the transport of blood and oxygen to the downstream tissue is often followed by reperfusion once the thrombus resolves either by itself or by means of medical intervention. This reperfusion is known to cause additional, so called reperfusional damage, with complement being known to play a role (162).

#### 2.1 C1q deficiency in stroke and neonatal hypoxic brain injury

A model of hypoxic ischemic brain injury in neonatal mice mimicking birth complications showed repeatedly a clear detrimental effect of C1q (48). Besides ligation of the carotid artery, the pups were exposed to hypoxia by 8% O_2_ over 15 min, as ligation alone does not reproducible cause tissue damage due to a pronounced collateral perfusion at this age. C1qKO mice presented with reduced infarction volume and better neurofunctional performance as compared to wt controls. Mitochondria in C1qKO mice had markedly reduced reactive oxygen species production even before the intervention, a difference that was no longer present in adulthood, pointing to a postnatally altered cell metabolism in the knockout model (48).

Results on brain ischemia in adult mice are less clear and show mostly little effect of C1q deficiency (49–51). Occlusion of the middle cerebral artery over 30-60 min followed by reperfusion caused slightly though non-significantly reduced infarct size in C1qKO mice as compared to wt controls (49). Application of C1 inhibitor (C1-INH) prior to occlusion reduced significantly infarct size, an effect even present in the C1qKO mice arguing strongly for a neuroprotective effect of C1-INH independent of the classical C1q-mediated complement pathway (49). Effects caused by binding of C1-INH to C3b or C4b seem plausible and match the finding of a protective effect of C3KO mice in stroke (50). Double knockout for the lectin and classical pathway protected from cerebral I/R injury (51). These data point to a dominant role of the lectin pathway. Similar to other settings, the study supports a critical amplification by the alternative pathway of an initial signal by the lectin or classical pathway for detrimental outcomes (51).

After acute ischemic injury of the retina, C1q deficiency prevented the loss of RGC and upregulation of microglia in retina as well as in the downstream superior colliculus (52). Retinal function was only initially rescued in C1qKO mice, but functional deficits did not differ by day 28 after I/R as compared to wt mice (52).

Thus, although prominently cited with respect to stroke models, the effects on infarct volume in neonatal studies (48) cannot be transferred to an I/R situation in adulthood (49–51) (**Table 1**). Aside from a different capacity for plastic changes, the neonatal brain has different metabolic capabilities and furthermore, as the authors point out, age dependent different functionality of complement pathways.

#### 2.2 C1q deficiency and I/R injury in other organs

I/R injury in organs other than the brain is most prominently affected by the lectin pathway rather than C1q and the classical pathway. MBL null mice were protected from myocardial infarction by temporary ligation of the left-anterior descending coronary artery, while C1qKO mice had unchanged left ventricular function and infarction size compared to wt controls (56). Similarly, MBL deficiency had a protective outcome with respect to muscle necrosis in hind limb ischemia (57) and skin necrosis area in cutaneous I/R (58). Here, C1q deficiency showed a similar trend which was not significant (58).

MBL is also a key player for I/R injury in gastrointestinal ischemia (53–55). In regard to C1q deficiency there was evidence for protection from I/R injury in male but not female mice (55), an effect which might have been missed in other studies (54). While C1qKO mice were protected against remote pulmonary injury as a sign of reperfusional damage in the severe protocol of 2 h of hindlimb ischemia (57), this was not the case in the setting of 20 min of gastrointestinal I/R (53).

### 3. C1q deficiency and liver diseases

C1q deficiency alleviated drug- or ethanol-induced hepatoxicity reducing hepatocellular apoptosis, inflammation and elevated liver enzymes in most cases (59–63). Protective effects of C1q deficiency on alcohol induced liver damage were modest overall. A detrimental effect of factor D deficiency was more pronounced and repeatedly shown, making factor D a protective factor (60, 62), and thus contrasting the commonly seen accelerating detrimental effect of alternative pathway activation. This may relate to high activation levels for adequate clearance of apoptotic cells or to the role of factor D as adipokine.

C1q deficient mice on a high fat diet as model for beginning non-alcoholic steatohepatitis became obese, but did not develop hepatic steatosis and insulin resistance pointing towards C1q as therapeutic target in obesity related glucose homeostasis derangements (63). Interestingly, there was no change in high fat diet-induced apoptosis of liver cells (63).

### 4. C1q and autoimmunity

#### 4.1 The generation of the C1q knockout mouse: a model for autoimmunity

The complement system and particularly the early components of the classical pathway appear to play a paradoxical role in SLE, the prototype of an autoimmune disease: While flares of SLE are associated with increased complement activity and consumption, inherited deficiency of C1q is a major risk factor for the development of SLE (1, 163). In order to study effects of C1qKO on autoimmunity, Botto et al. created the C1qKO mouse in 1998 (9). In line with the clinical presentation of hereditary C1q deficiency in humans, C1q deficient mice developed anti-nuclear antibodies (ANA) in 55% of cases and glomerulonephritis (GN) in 25% of cases (9), features and distributions with remarkable similarity to human SLE. One striking finding was the high number of apoptotic cell bodies and blebs in the kidneys of C1qKO mice, which was present even in the absence of the development of GN. This fostered the *waste disposal hypothesis* as one – though not exclusive – explanation for the development of systemic autoimmunity (see 4.4) (164–166). Notably, already in the first impressive and very thorough description of the C1qKO mice, it became clear that the development of autoimmunity was dependent upon two aspects: genetic background and gender (9).

#### 4.2 C1q deficiency as disease accelerator in the presence of lupus prone background genes

C1q deficiency itself does not necessarily cause autoimmunity as neither mice with a pure 129/Ola background (9) nor C57BL/6 mice (71) developed auto-antibodies. Only in the presence of a permissive lupus-prone genetic background, C1q deficiency caused accelerated autoimmunity. As mentioned above, the initial description by Botto et al. clearly pointed out that only mice on a mixed genetic background, namely the F2 generation 129/Ola×C57BL/6, showed substantial autoantibodies and GN, while none of the pure 129/Ola background mice developed GN (9). Following up on this finding, Bygrave et al. could show that a 129-derived segment on chromosome 1 in a B6 background was sufficient to have profound effects on autoimmunity leading to high ANA titres (167), thus showing that 129/Ola×C57BL/6 itself is a susceptible genetic background for autoimmunity. Notably, 129/Ola×C57BL/6 mice are widely employed to generate gene-targeted mice. The fundamental assumption underlying the investigation of knockout-models, namely that the null-gene is causally related to disease outcome, is therefore heavily confounded in complement knockout mice with respect to autoimmunity. In the presence of C1q deficiency, the 129-derived region on chromosome 1, as well as a region on chromosome 3 from the B6 could be confirmed to be linked to ANA expression (73). Similarly, regions on chromosome 7 of 129 and chromosome 13 of B6 mice were strongly linked to GN (73).

C1q deficiency accelerated the development of autoimmunity not only on a 129/Ola×C57BL/6 mixed genetic background but also on other lupus-prone genetic backgrounds. While in the setting of a nonpermissive background of C57BL/6 mice C1q deficiency did not induce autoimmunity, it did lead to an accelerated progress of disease in lupus prone MRL/Mp^+/+^ animals (71). MRL/Mp^+/+^ mice are the parent strain of the better known MRL/Mp-*lpr/lpr* mice but with intact *Fas* gene, nevertheless known to be prone to autoimmune features (168). When introducing a *Fas* deficiency by *lpr* mutation, disease acceleration by C1q deficiency did not occur. This is possibly due to the advanced severity of the disease in C57BL/6.*lpr/lpr* and MRL/Mp-*lpr/lpr* strains, which may not have allowed for the detection of accelerating effects (71).

C1q deficiency could be overcome by bone marrow transplantation, proving the monocyte/macrophage lineage as major source of C1q and at the same time offering a potential treatment option for SLE in C1q deficient patients (69, 169). In addition, bone marrow transplantation alleviated the autoimmune phenotype on a lupus prone MRL/Mp background (72). This study provided clear evidence for a causality of C1q deficiency for the observed autoimmunity.

Thus, it is not C1q deficiency *per se* that relates to autoimmunity. Rather C1q deficiency has the ability to accelerate autoimmune disease, when a lupus prone genetic background with a high tendency for autoimmunity is present (**Table 2**).

#### 4.3 Gender affects autoimmune manifestations in C1qKO mice

In addition, an effect of gender on the development of autoimmunity was described by Botto et al.: When comparing C1q sufficient mixed background controls to C1q deficient mice, a significant difference in ANA levels only existed between males. Control females on the mixed background already started off with high titres. An increased susceptibility to manifestation of autoimmunity in females was confirmed in double knockout mice for C1q and H2-Bf/C2^-/-^ (80) as well as C1q deficiency in the lupus prone MRL/Mp background (71). Although this effect is particularly well described for autoimmunity on a lupus prone genetic background, it is still noteworthy that a large number of studies in C1qKO mice included in this review investigated female mice only (4, 11, 38, 59, 60, 62, 73, 76–79, 87, 97, 116, 125, 128, 146, 149, 170) while others only used male animals (26, 35–37, 45, 46, 50, 53, 61, 63, 88, 99, 110, 113, 119, 141) (**Tables 1**–**3**). Few studies evaluated specifically the differential gender effect (12, 24, 28, 55, 71, 80).

The gender specificity of autoimmunity is particularly exciting from a translational approach as human autoimmunity *per se* and SLE in particular clinically shows a clear gender effect with predominantly young female patients being affected (171).

#### 4.4 Using murine C1q deficiency to understand underlying mechanisms of autoimmunity

Autoimmunity is characterized by auto-antibodies directed against self-antigens with the underlying mechanisms as to why autoimmunity arises in certain patients but also in certain mouse models being still a matter of debate. In the setting of C1q deficiency the striking amount of apoptotic cells (9), the fact that autoantibodies directed against proteins contained in apoptotic blebs as well as the well accepted role of C1q in the removal of cell debris has led to the *waste disposal hypothesis*: a prolonged exposure of the body to cell debris fuels antibody formation (164, 166, 172). Besides high numbers of apoptotic cells in kidneys with and without GN (9, 80), the rate of clearance of apoptotic cells was also reduced in C1qKO mice in a model of sterile peritonitis (66). While the involvement of C1q in the clearance of apoptotic cells is a robust finding and widely accepted, removal of apoptotic cells and induction of autoantibodies and GN were unchanged when apoptosis was induced by ultraviolet light in an attempt to mimic a sunburn-triggered SLE flare (70), indicating that the effect cannot be generalized to all tissues and disease settings. One study pointed to mainly lysed cells, such as those in necrotic tissue, as aggravating agent of autoimmunity in C1qKO mice, while intact apoptotic cells did have no effect (65).

Another – and not mutually exclusive –hypothesis on the role of C1q deficiency in the development of autoimmunity is a modulating influence of C1q on B and T cell autoreactivity (67, 77). The results of C1q deficiency on B cell autoreactivity were inconsistent ranging from increased positive selection of auto reactive B cells (74) to no clear effect (64, 67, 75). While effects on B cells are inconsistent, it is apparent that C1q deficiency has effects on T-cell responses and related cytokine levels as well as on dendritic cell antigen processing in the spleen (67, 77, 79, 173). A more recent comprehensive publication focused on the question as to why C1q but not C3 deficiency is critical for self-tolerance, showing that C1q specifically altered mitochondrial metabolism of T-cells (77). C1q deficiency led to a skew towards an effector CD8+ T-cell phenotype in response to chronic viral infection (77). Thus, self-tolerance in C1q deficiency may be lost due to an inadequate CD8+ T cell response to viral infection (77). An earlier report described chronically accelerated CD4+ T-cell activation and splenic monocytosis caused by C1q deficiency in a lupus prone genetic background (79). In addition, C1q was tightly linked to processing of immune complexes with splenic uptake of immune complexes being significantly reduced in C1q deficient mice (64, 68). Beyond effects on specific immune cells other mechanisms may also be of relevance. Molecular mimicry could be a link between epstein-barr virus infections and the formation of anti-C1q antibodies in SLE as shown in C1qKO mice (78).

In contrast to the great number of studies using C1q deficiency in lupus prone backgrounds to induce or accelerate autoimmunity, there is a single report showing alleviated autoimmunity in C1q deficiency (**Table 2**): when the SLE phenotype was induced by the i.p. injection of the oily substance pristane (76). In this setting, C1q deficiency surprisingly reduced auto-antibody titers and caused milder arthritis (76).

#### 4.5 C1q deficiency in autoimmune nephropathies

##### 4.5.1 Lupus nephritis is a typical feature in C1qKO mice

Lupus nephritis (LN) is a typical feature in C1qKO mice on a mixed genetic background (9, 80). LN is characterized by immune complex formation containing deoxyribonucleic acid (DNA) and anti-ds DNA immune complexes as well as C1q, laminin and other auto-antibody targets. While C1q deficiency was associated with an increase of apoptotic cells in kidneys, clearance did not require C3 activation (80). C1q deficiency also acted as disease accelerator in a polygenetic model of LN in which low complement was mimicked by C1q deficiency, autoantibody formation was induced by *Sle1* knockout (*Sle1*-KO) and defective clearance of apoptotic cells by *Mfge8* knockout (*Mfge8-*KO*)* (81). There was, however, no significant effect when comparing C1q sufficient *Sle1*-KO with C1q deficient *Sle1*-KO mice. In addition, anti-C1q antibodies are strongly associated with LN. In a model of LN by application of anti-glomerular basement membrane (GBM) antibodies as well as mouse anti-C1q antibodies, C1qKO mice did not show increased albuminuria (82), indicating that glomerular C1q-containing immune complexes are essential for disease manifestation in this setting.

##### 4.5.2 Other autoimmune nephropathies

There are a variety of other forms of autoimmune nephropathies. Models of anti-GBM glomerulonephritis were overall aggravated by C1q deficiency (83–85) (**Table 2**). Anti-GBM antibody application led to severe glomerular thrombosis within four days of induction of disease in C1q-deficient mice compared to mild injury in wt controls (83). Again, C1q deficiency was only associated with increased nephritis susceptibility on a mixed genetic background but not on a pure C57BL/6 background (84, 85). Differences in susceptibility to glomerular inflammation were indeed so apparent in the investigated strains, that it could not be excluded, that the effect was caused by the background genes rather than the absence or presence of C1q (84).

Immune-complex glomerulonephritis induced by cryoglobulins remained unaltered by C1q deficiency (86). Furthermore, models which investigated sclerosis and fibrosis of the kidney remained unaffected by C1q deficiency (87, 88).

In summary, while in lupus prone strains C1q deficiency readily accelerates lupus nephritis (9, 80, 81), other forms of immune complex glomerulonephritis are not necessarily affected by C1q deficiency (86). Similarly, immune mediated nephritis induced by injection of anti-glomerular antibodies is deteriorated in certain mice strains but not on a non-permissive C57BL/6 background suggesting that the sole absence of C1q is not sufficient to cause disease but requires additional genetic and/or environmental factors (83–85).

#### 4.6 C1q deficiency and tolerance induction in organ transplantation

Transplant rejection is associated with C3 deposition and related to complement activation (174). Counterintuitively, C1q as well as C3 appear to be protective with respect to allograft rejection as demonstrated by an earlier rejection of a solid organ transplant in C1qKO as well as C3KO mice (89–91). Intranasal tolerance induction failed in complement (C1q and C3) deficient mice (90). Importantly, models addressing T-cell specific graft responses (HY mismatched skin graft) showed faster rejection in C1q deficiency (90). Accepted grafted skin had increased mRNA levels of C1q (as well as C3 and interferon -γ), making an argument for a contribution of C1q to tolerance induction also with respect to grafted tissue (90).

#### 4.7 C1q deficiency and arthritis

C1q deficiency alone did not change the outcome in a model of inflammatory arthritis induced by anti-collagen antibodies, instead the alternative pathway was responsible for driving the disease (92, 93). While factor D deficiency protected against disease manifestation, additional C1q deficiency did not result in further benefit (93).

#### 4.8 C1q deficiency and vaccination

In models of vaccination (95, 97) and gene therapy vectors (98, 99), there was no major effect of C1q deficiency. Immunoprophylaxis by alloimmunization as performed e.g. in rhesus prophylaxis failed when performed at a timepoint of concomitant viral infection. This failure of immunoprophylaxis was complement dependent and C1qKO mice expressed successful alloimmunization under concomitant inflammation (96).

### 5. C1q deficiency and Infections

#### 5.1 C1q deficiency aggravates bacterial infections

Complement is a cornerstone of innate immunity, giving rise to a rapid response to a number of pathogens. About 40% of patients with C1q deficiency present clinically with recurring severe bacterial infections (1). In agreement, the outcome of C1q deficient mice in studies on bacterial infections was exclusively negative (**Table 2**).

The encapsulated gram-positive diplococcus *Streptococcus pneumonia*, numerously studied in C1qKO mice, is the most common pathogen in human bacterial pneumonia and a highly relevant pathogen regarding meningitis (102–105, 108). In all investigated modes of infection (i.p., intranasal, intravenous, transtympal, intracysternal) C1q deficient mice presented with higher pathogen titers in tissue samples (102, 104–106, 108). Complement C3 and C1q protected against the spread of *Streptococcus pneumonia* into the blood, an effect which was even more pronounced in C3 deficient mice (104, 105). C1q deficiency (as well as factor B deficiency) reduced opsonization of *Streptococcus pneumoniae* in a model of otitis media (107) and phagocytosis triggered by activation of the classical pathway *via* IgM appeared to be the dominant route of defense against *Streptococcus pneumoniae* (102, 106). Protection against pneumococcal infections in a passive immunization model was dependent upon classical pathway activation, but not on a functional leukocytal Fc gamma receptor (103). The bacterium has developed specific virulence factors against complement like pneumolysin, which is expressed in certain strains of *Streptococcus pneumonia* and binds C1q (104). Counter intuitively, pneumolysin rather acted by activating than inhibiting the classical pathway, but the relation to decreased complement deposition on the bacterium remained unclear (104).

Although, it is the alternative pathway that is essential for the clearance of *Streptococcus pyogenes* infections, C1qKO mice also readily succumbed in *Streptococcus pyogenes* septicaemie due to inefficient phagocytosis (109). Similarly, C1qKO mice undergoing polymicrobial infection had greatly heightened mortality (94, 112), which was even inducible by low pathogen loads (110).

Many gram-negative bacteria are not susceptible to complement attacks due to the expression of complement regulators. However, possibly due to disturbed phagocytosis, C1qKO mice were unable to limit *Salmonella enterica* serovar Typhimurium growth with animal deaths starting to occur 6 days after transfection (113). Also infections with *Borrelia burgdorferi*, a tick transmissible spirochete, caused higher pathogen load in C1q deficiency, though clinical effects were comparatively mild (114). In this case, the pathomechanisms may relate to a delayed IgG class switch, altered T-cell response, and altered cytokine levels (114). Similarly, the pathogen burden in various organs during infections with *Rickettsia*, yet another intracellular pathogen transmitted among others by ticks, was significantly increased in C1qKO mice (115).

#### 5.2 C1q deficiency and new antibacterial therapies

New antibacterial therapies against the increasingly drug-resistant *Neisseria gonorrhoeae*, a pathogen causing the sexually transmitted disease gonorrhoe, use chimeric antibodies with increased capability to form hexamers targeting specifically C1q and the complement pathway (116, 117), as well as other C1q independent virulence mechanism like sialylation of *Neisseria gonorrhoeae* lipooligosaccharides (118). The prophagocytic therapeutic effect of the pattern recognition receptor PTX3 against *Pseudomonas aerruginosa*, a pathogen associated with chronic lung infections, was similarly unchanged in C1qKO mice (119).

#### 5.3 C1q deficiency and infections with other pathogens

The investigation of pathogens other than bacteria yielded mainly no effect of C1q deficiency on clinical outcome (122, 124) with the exceptions of negative outcomes in the infection with the malaria causing parasite *Plasmodium chabaudi chabaudi* (121) and the viral infection with West Nile virus (120) (**Table 2**). High viremia and mortality in C1qKO mice with spleens remaining virtually free of infectious virus again underpins the crucial role of C1q in transport and pathogen processing in this lymphoid organ (120, 121).

### 6. C1q deficiency and vessels

Atherosclerosis is a disease of the arterial vessel wall with a complex pathogenesis involving endothelial dysfunction, inflammatory, and immunological processes accompanying plaque formation. Apoptotic cells, while efficiently removed by activated macrophages in early lesions, are prominent in late lesions. Genetic knockout of low-density lipoproteinreceptor (LdlrKO) serves as a model of atherosclerosis. Lesion size and complexity is increased by additional C1q deficiency in LdlrKO mice kept on a high fat diet (125). IgM-Ldlr double knockout mice had even greater lesions, even when compared to triple knockout mice lacking IgM, Ldlr, and C1q (126). This observation supports IgM tagging of cholesterol with subsequent C1q-stimulated removal by macrophages as an important pathomechanism.

Angiogenesis, the formation of new vessels, is a tightly regulated mechanism physiologically occurring e.g. during wound healing. In skin wounds, vessel formation was found to be insufficient in C1qKO mice and could be restored upon the local application of C1q, suggesting a role of C1q as angiogenic factor for the treatment of chronic ulcera (127). C1qKO mice expressed altered lung vascular homeostasis with enhanced susceptibility of the pulmonary endothelium in response to injury (128).

Finally, the complement and coagulation cascades have been shown to interact at various levels (175) including the interaction of the two initiators von Willebrand factor and C1q (176, 177). The functional relevance of C1q in primary hemostasis was demonstrated by prolonged bleeding time of C1qKO mice in tail bleeding experiments (129).

### 7. C1q deficiency and pregnancy

C1q is present at the feto-maternal interface and has a promoting role for trophoblast invasion of the decidua (178). C1q deficiency was associated with smaller litter size, reduced fetal weight and increased frequency of fetal resorption (130). However, C1q deficiency is not related to fetal loss *per se* as there was no influence on fetal loss in a model of dysregulated uterine conditions induced by Il-2 primed T-cells directed against paternal antigens (132). Interestingly, litter size and fetal resorption was also not affected by C1q deficiency as compared to wt mice in this study (132), contradicting earlier findings (130).

Double knockout mice for C1q and factor D, i.e. defective classical and alternative pathways, were protected from fetal loss induced by antiphospholipid syndrome, a well-known clinical cause for miscarriage (131). This detrimental role of complement activation in fetal loss in antiphospholipid syndrome was further supported by robust C4 deposition at feto-maternal interface as well as in human tissue from SLE and/or antiphospholipid syndrome patients (131).

A robust and impressive phenotype of C1q deficiency is the development of preeclampsia (PE) characterized by hypertension, albuminuria, glomerular endotheliosis, and decreased levels of placental vascular endothelial growth factor (133–135). Development of PE relates to C1q deficiency of the fetus rather than the mother as it is also observed in C1q competent females carrying offspring from C1q deficient males (135). As in other models of PE, high-dose pravastatin treatment (amounts of 5 mg per day compared to 20-40 mg per day dosage commonly used in humans as lipid-lowering agent) could obviate the condition and restore trophoblast invasiveness (135). PE caused persisting endothelial dysfunction for up to 6 months in C1q sufficient mothers and offspring as well as microglia activation in offspring, all of which responded to pravastatin treatment (133, 134).

### 8. C1q deficiency and cancer

C1q deficiency yielded conflicting effects in different cancer models (136–138) (**Table 3**). In a breast cancer model of Her2/neu transgenic (neuT) mice, C1q deficiency was associated with accelerated formation of lung metastases and intratumoral vessel formation, arguing for C1q as inhibitor of tumor angiogenesis (137). In contrast, a detailed study on implanted melanoma cells showed lower vascular density and fewer metastases as well as slowed down tumor growth and prolonged survival of C1q deficient mice (136). Human tissue expressed high levels of C1q in the stromal parenchyma at the tumor invading zone, arguing for C1q as a locally derived tumor promoting factor. Similarly, tumor vascularization was impaired in C1qKO mice in a model of lung carcinoma cells (138). Of note, the lung tumor cells were inoculated subcutaneously and thus in a similar microenvironment as the melanoma cells in the study by Bulla et al. (136). These results are not easy to reconcile with the breast cancer study (137). Besides a peculiar role of the skin as microenvironment for tumor growth or the effect of different background genes (BABLc versus B6) it is very likely that different cancer entities do not have comparable pathomechanisms of progression.

With respect to cancer treatment, B-cell depletion by the chimeric CD20 antibody rituximab appears to be critically dependent upon the presence of C1q (139), while mouse anti-CD20 antibody acted independently of C1q (140), a discrepancy which may relate to a species specificity of the used Fc segment. Unwanted toxic effects by high cytokine levels when using co-stimulatory small molecules were not affected by C1q deficiency (141). The synergistic effect of the combinatory therapy with trastuzumab and pertuzumab in hormone-receptor positive breast cancer was abolished in C1qKO mice (142).

### 9. Areas with limited evidence

In some disease models there are currently only singular studies employing C1qKO mice. Areas with beneficial effects of C1q deficiency include muscle regeneration in aged mice (145), sterile inflammation by i.p. administration of polyglycolic acid as present in degradation processes of absorbable sutures (101). Epidermolysis bullosa acquisita with subepidermal blisters induced by antibodies directed against collagen VII was most prominently influenced by the alternative pathway, while C1q deficiency only decreased the extent of skin disease at the end of the observation period (144).

Deleterious effects in C1q/MBL double-deficient mice were seen in inflammatory bowel disease modeled by dextran sulfate sodium-induced colitis (100). Effects of C1q on regulatory T cell differentiation also are related to a marked increase in lung inflammation in a mouse model for smoke induced chronic obstructive pulmonary disease (146). While the number of immune cells is unchanged in a model of silicate induced lung fibrosis, C1q deficiency was beneficial with respect to fibrotic changes (147). Conversely, intratracheal application of C1q in wt mice induced fibrotic changes (147).

In models of anaphylaxis (94), eschar formation after burn injury (143), and amyloidosis (148) there was no major effect of C1q deficiency. Inflammatory cytokines expressed in adipocytes in response to high ethanol feeding were reduced in C1q deficient mice with the number of apoptotic adipocytes remaining unchanged (149).

## Discussion

### C1q as target molecule to treat human disease

**Figure 2:**
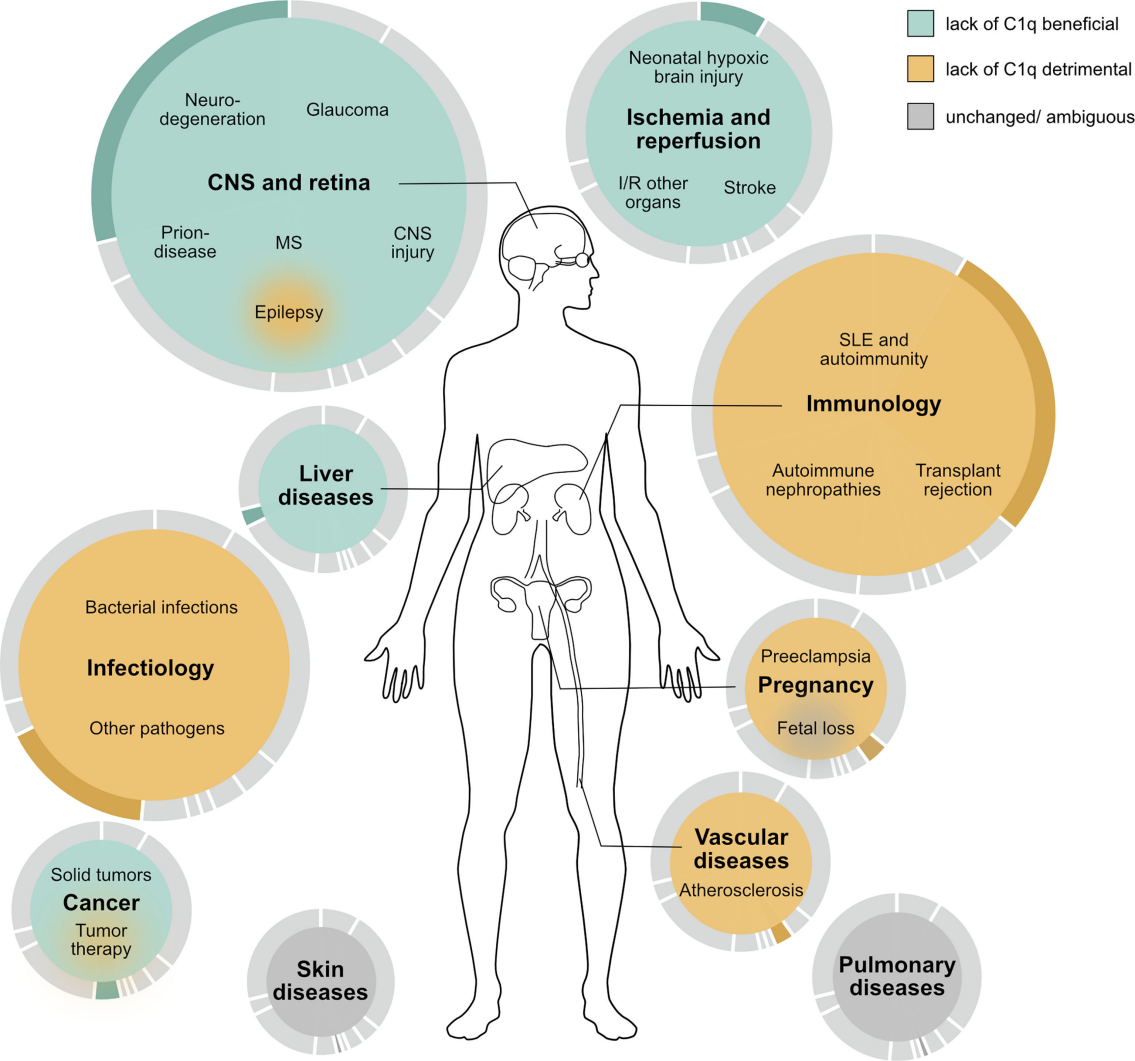
Schematic overview of the proposed effect of C1q deficiency on human disease based on the outcome of studies performed in murine disease models. Beneficial effects are colored in turquoise, detrimental in ocher, and outcomes without general trend or with ambiguous results in grey. The size of the colored area and the underlying pie chart illustrate the number of studies performed in the respective area. MS multiple sclerosis, I/R ischemia and reperfusion, SLE systemic lupus erythematosus.

Are there diseases where C1q is harmful and a potential target protein to treat certain diseases? From an evolutionary perspective any protein in our body serves a purpose and can therefore in the first place be regarded as beneficial. In the case of C1q, this view is further underpinned by the evolutionarily conserved presence of C1q in many species. Clearly, when looking at immune defense and autoimmunity, C1q is not only beneficial but even critical. However, there may be circumstances where the presence of C1q becomes detrimental and the absence of C1q such as in the C1qKO mouse is beneficial. In the presented overview of disease-focused studies employing the C1qKO mouse, we identified neurodegenerative diseases, including glaucoma and secondary neurodegenerative processes after TBI, postexposure prophylaxis in prion disease and drug-induced liver inflammation as the most promising settings, in which an inhibition of C1q might be therapeutically valuable (**Figure 2**).

Most diseases with beneficial outcome in C1q deficiency can be summed up under the term sterile inflammation, while septic inflammation is associated with detrimental outcome in C1qKO mice. C1qKO mice had a beneficial outcome in response to application of substances (61), ethanol (60, 131), suture material (101) or amyloid plaques in neurodegeneration (2, 19). Even pristane induced SLE might be regarded in this context (76). In contrast to sterile inflammation, septic conditions caused by pathogens required the presence of C1q as seen by the detrimental outcomes of the C1qKO mouse (102–106, 108–114) (**Table 2**). An exception to this rule is prion disease (3, 4), where C1q blockage may have great potential in postexposure prophylaxis.

Experimentally, C1q-blocking antibodies have been applied in a number of murine studies in recent years with promising results. Mainly neurological disease entities were tackled with main effects being shown on microglia: from potential synaptic rescue in transgenic Tau-P301S AD model mice (179) as well as wt mice challenged with intraventricular injection of soluble amyloid *β* oligomers (21) and neurolupus (180) to effects on microglia as a therapeutic approach to reduce chronic white matter inflammation in MS (44) and TBI (34). In mild TBI, C1q antibody application reduced secondary inflammatory neurodegeneration and protected against sleep pattern disruption and epileptogenic potentials (34). Interestingly, the i.p. application caused complete depletion of plasma C1q, while C1q levels in brain-homogenate − though significantly reduced − were detectable and remained above half of the control level, possibly also due to intracellular mostly microglial derived protein (34). Indeed, a number of clinical trials in humans have been undertaken to treat neurological as well as autoimmune diseases by delivering an anti-C1q antibody, which depletes and/or blocks serum C1q (181, 182). Currently these trials are in phase 2 for Guillain-Barré Syndrome, warm autoimmune hemolytic anemia, Huntington’s disease and ALS (182). While treatment of Guillan-Barré Syndrome will be performed over a limited time period and a conference abstract reported promising results of a Phase 1b study on single-ascending-dose with respect to safety but also outcome (183), in the setting of Huntington’s disease long term treatment might be necessary.

Based on the presented studies in animal models, there are several challenges arising when considering the treatment of diseases with anti-C1q directed antibodies. First, most relevant diseases would require treatment early on in the course of disease. This holds particularly true for glaucoma, one of the most promising candidates. Based on experimental data, anti-C1q blocking treatment might be required even before axonal damage and thus before symptom onset (12). Also with respect to neurodegenerative AD there is evidence for C1q being an early mediator of synapse loss (21). Second, treatment may be required over extended periods of time particularly in chronic diseases such as neurodegeneration and glaucoma. Third, a systemic blockage or depletion of C1q as a treatment could cause severe side effects, particularly if treatment is required over extended time periods. The main side effects to be expected under C1q depletion encompass elevated risk of infections, endangered pregnancy, and development of autoimmune phenomena. The outcome of C1qKO mice in models of bacterial infections is pronounced (**Table 2**) and the immuno-suppressive effects of C1q depletion over longer time periods is likely to be relevant. When thinking of anti-C1q antibodies as a treatment for MS, as recently demonstrated in mice (44), the effects on pregnancy in this patient group need to be carefully considered. Autoimmunity in mice heavily depends on background genes in whose presence C1q deficiency may act as a disease accelerator. Possibly, only a limited number of individuals may be affected by autoimmune phenomena in response to prolonged systemic C1q inhibition. Nevertheless, the effect of C1q deficiency on autoimmunity seems to be more pronounced in human beings than in mice with about 77% of individuals with C1q deficiency developing SLE or SLE-like disease. In addition, anti-C1q antibodies targeted to the collagen-tail can aggravate immune complex nephritis in mice (82) and similarly therapeutic agents could aggravate ongoing immune processes in patients.

Current approaches use anti-C1q antibodies, which seem to cause a serum-depletion of C1q (181). This depletion, while being highly effective, is nevertheless the key to expected side effects. C1q is a highly functional and large molecule which poses the question of whether we can make use of its functionality. It is not unlikely that C1q expresses more than one functional domain that consequently mediates different downstream effects. There is the possibility, that specific cryptic epitopes relate to particular diseases and/or symptoms (184). Cryptic epitopes are hidden and only become exposed upon e.g. binding of specific target structures. A better understanding of these epitopes as well as downstream receptors and pathways would allow us to target therapies more precisely and avoid major side effects from unspecific depletion of this highly versatile molecule.

### Limitations

The usage of C1qKO mice is an attractive tool to explore the causal relevance of the protein in *in vivo* models of disease. A mere correlation of C1q levels with disease manifestation may be misleading and unchanged disease manifestation in C1qKO mice can reveal missing causality (14, 43).

Though C1q knockout appears to be a very clean and clear-cut method to address the role of C1q, there are potential drawbacks to be considered. An evolutionarily conserved and highly relevant system like the complement cascade is bound to rely on multiple routes rather than on a single pathway/protein. Thus, it is possible that compensatory mechanisms come into play in the C1qKO mouse. The interactions of complement proteins are not as linear as often depicted on overview figures such that knocking out one player may have unexpected effects on other pathways (27). C1q might even have opposing effects on different mechanisms within a specific disease (20). Furthermore, when going into technical details it becomes clear, that the target gene cannot be knocked out without having some residual genetic information remaining. Specific 129 derived intervals on chromosome 1 were sufficient to have profound effects on autoimmunity namely the loss of tolerance to nuclear antigens (167), thus creating a major confounder. It is not always given, that the null-gene is exclusively causally related to disease outcome.

Second, there is the challenge of translating results from the mouse to the human, and vice versa modelling a human disease in a murine model. Disease models in mice are limited in various ways. In order to create a disease model, one must make certain assumptions on the causal origin of the disease to best mimic the disease. These assumptions might be misleading. Alternatively, the close observation of certain symptom sets in mice resembling features of human disease may lead to establishing a disease model. However, while symptoms may be similar, underlying pathomechanisms do not need to be. In addition, species differences like certain immunological features or levels of complement components may change pathomechanisms and hamper the transfer of murine outcomes into the human disease setting. Additionally, some diseases or symptoms may be utterly impossible to investigate in murine models e.g. aphasia in FTDL.

Finally, there are limitations in the design of this review. First, while we used broad search terms in our database query it is possible that we missed some publications. Second, negative results are not as regularly published as studies which are able to show an effect leading to a publication bias. Third, not all studies were designed to explore the outcome of C1qKO versus wt mice, which particularly limits the interpretation of studies using C1qKO in combination with another gene knockout.

## Concluding remarks

The specific understanding of pathomechanisms in diseases involving the complement system has enabled us to apply targeted treatment. An example is paroxysmal nocturnal hemoglobinuria, which is caused by deficiency in CD55 and CD59, making red blood cells susceptible to complement-mediated lysis. Nowadays, paroxysmal nocturnal hemoglobinuria can be successfully treated with the anti-C5 antibody eculizumab. C1q might be another upcoming target worthwhile exploring to treat or prevent for example neurodegenerative diseases, a disease entity with an immense socio-economic burden.

Several publications cited here made attempts to define good and bad players of the complement system. A quote from Benhar et al. seems applicable not only with respect to immune cells but all players of the immune-system: *“ there are no ‘good’ or ‘bad’ immune cells; it is all a matter of their control and coordination “* (185). Indeed, it would make little sense from a biological perspective to have molecules or cells, which are under all circumstances “bad guys”. As with anything in nature, it is often about maintaining the balance. This systematic review contributes to identifying those diseases, where C1q might be out of balance. First approaches today use anti-C1q antibodies, which seem to cause a serum-depletion and/or blocking of C1q (181). It is conceivable that a more specific targeting of specific epitopes of C1q may result in a therapeutic outcome with reduced systemic side effects. Future translational research is needed to evaluate adequate approaches to re-establish an equilibrium without causing side effects of the other extreme.

## Data availability statement

The original contributions presented in the study are included in the article/**Supplementary Material**. Further inquiries can be directed to the corresponding author.

## Author contributions

KS: drafting, conceptualizing, and editing. MT: conceptualization, reviewing, editing, and supervision. All authors contributed to the article and approved the submitted version.

## Funding

MT is supported by a project grant of the Swiss National Science Foundation (SNSF) (320030_200423/1). KS is supported by the reglementary pool of the Division of Internal Medicine, University Hospital, Basel, Switzerland.

## Acknowledgments

We thank Pascal Rabatscher, Claudia Donat and Eylül Tuncer for valuable input to the manuscript. We thank Shakuntala Savanthrapadian and Patricia Dietrich for editing.

## Conflict of interest

The authors declare that the research was conducted in the absence of any commercial or financial relationships that could be construed as a potential conflict of interest.

## Publisher’s note

All claims expressed in this article are solely those of the authors and do not necessarily represent those of their affiliated organizations, or those of the publisher, the editors and the reviewers. Any product that may be evaluated in this article, or claim that may be made by its manufacturer, is not guaranteed or endorsed by the publisher.

## Abbreviations

AD, Alzheimer’s disease; ALS, amyotrophic lateral sclerosis; AMD, age-related macular degeneration; ANA, anti-nuclear antibody; APP, amyloid precursor protein; CNS, central nervous system; dLGN, dorsolateral geniculate nucleus; DNA, deoxyribonucleic acid; EEG, electroencephalography; EAE, experimental autoimmune encephalomyelitis; FLTD, frontotemporal lobar degeneration; GBM, glomerular basement membrane; GN, Glomerulonephritis; GRN, progranulin; GRP6, G protein-coupled receptor 6; I/R, ischemia/reperfusion; LN, lupus nephritis; LRP1B, lipoprotein receptor-related protein 1B; Ldlr, low-density lipoproteinreceptor; MAG, myelin associated glycoprotein; MBL, mannose-binding lectin; MOG, myelin oligodendrocyte glycoprotein; MPTP, 1-methyl-4-phenyl-1,2,3,6-tetrahydropyridine; MS, Multiple sclerosis; PE, preeclampsia; PFC, prefrontal cortex; PNS, peripheral nervous system; PrP^c^, cellular prion protein; RGC, retinal ganglion cells; SCID, severe combined immunodeficiency disease; SLE, systemic lupus erythematosus; SOD1, superoxide dismutase 1; TBI, traumatic brain injury; TTR, transthyretin; TNF, tumor necrosis factor; wt, wild type.
