# Supplementary material for: C1q as a target molecule to treat human disease: What do mouse studies teach us?
Source: Front Immunol. 2022 Aug 3;13:958273. doi: 10.3389/fimmu.2022.958273 (PMC9385197; doi:10.3389/fimmu.2022.958273)
Supplement: Supplementary file 1 [file DataSheet_1.docx]

*Supplementary file 1: detailed search strategies*

**PubMed search performed on 22nd April 2022**:

(("complement c1q"[MeSH Terms] OR ("complement"[All Fields] AND "c1q"[All Fields])

OR "complement c1q"[All Fields]) AND ("mice"[MeSH Terms] OR "mice"[All Fields] OR "mouse"[All Fields] OR "mouse s"[All Fields] OR "mouses"[All Fields])) AND (1998:2022[pdat])

**EMBASE search performed on 22nd April 2022**:

('complement c1q'/exp OR 'complement c1q' OR (('complement'/exp OR complement) AND ('c1q'/exp OR c1q))) AND ('mouse'/exp OR mouse) AND (1998:py OR 1999:py OR 2000:py OR 2001:py OR 2002:py OR 2003:py OR 2004:py OR 2005:py OR 2006:py OR 2007:py OR 2008:py OR 2009:py OR 2010:py OR 2011:py OR 2012:py OR 2013:py OR 2014:py OR 2015:py OR 2016:py OR 2017:py OR 2018:py OR 2019:py OR 2020:py OR 2021:py OR 2022:py) AND ('Article'/it)
